# Isolated Testicular Metastasis Diagnosed More than a Decade and a Half Post Primary Treatment for Prostate Cancer

**DOI:** 10.1155/2019/4956954

**Published:** 2019-11-26

**Authors:** Nassib F. Abou Heidar, Gerges Bustros, Jose M. El-Asmar, Bassel Zein Sabatto, Jad A. Degheili

**Affiliations:** ^1^Division of Urology, Department of Surgery, American University of Beirut-Medical Center, Riad El-Solh, 1107 2020 Beirut, Lebanon; ^2^Department of Pathology and Laboratory Medicine, American University of Beirut-Medical Center, Riad El-Solh, 1107 2020 Beirut, Lebanon

## Abstract

Prostate cancer is the most common visceral malignancy among men. It rarely metastasizes to the testicles. We herein present the case of a male patient who underwent a radical prostatectomy for a grade group 3 Gleason score 7 (4 + 3) prostate adenocarcinoma followed by adjuvant radiation therapy and continuous androgen deprivation therapy after his first biochemical recurrence. Despite optimal management, prostate-specific antigen (PSA) levels rose back up, upon which a PET/CT ^68^Gallium scan demonstrated an isolated left testicular lesion that turned out to be of prostatic origin following orchiectomy. Testicular metastases from prostate cancer are of unknown prognosis, and the current treatment modality favors an orchiectomy.

## 1. Introduction

Prostate cancer (PCa) is the most common noncutaneous malignancy in men; the most common sites of metastasis include lymph nodes, bones, lungs, and liver [1]. Testicular metastasis from prostate cancer is seldom reported in literature and occurs in up to 4% of cases. It is mostly an incidental finding upon pathological studies following orchiectomy for surgical castration [2] or following postmortem autopsies. We hereby present a distinctive case of prostate cancer with left testicular metastasis, 16 years after definitive management for prostate cancer by an open radical prostatectomy. To the best of our knowledge, this is only the 12^th^ case to be reported.

## 2. Case Presentation

A 79-year-old male was diagnosed with prostate cancer in 2003 after a screening prostate-specific antigen (PSA) level of 7 ng/mL, alongside a firm prostate upon digital rectal examination. A transrectal ultrasound (TRUS) guided biopsy at the time revealed a Gleason score of 7 (4 + 3) adenocarcinoma of the prostate. He underwent an open retropubic radical prostatectomy with bilateral pelvic lymph node dissection. The final pathology result revealed a Gleason score of 7 (4 + 3) adenocarcinoma with prostatic capsule and seminal vesicle involvement as well as perineural invasion. Studies were consistent with a pT3bN0M0R0 disease; surgical margins were negative. Postsurgical PSA dropped to a nadir of less than 0.02 ng/mL.

**Figure 1:**
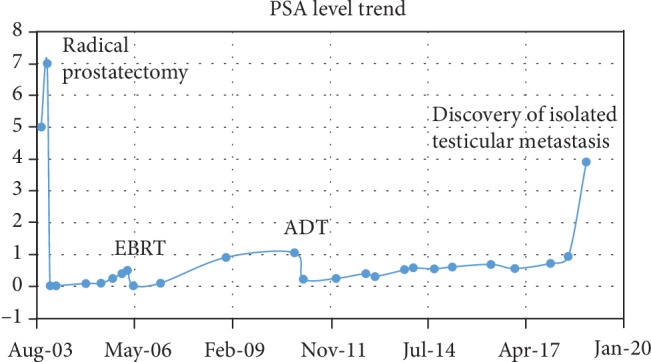
Trending of PSA levels over the 16-year time span, from the diagnosis of prostate cancer to the discovery of isolated testicular metastasis.

Three years after his surgery, his PSA level gradually rose to 0.5 ng/mL for which he was started on adjuvant external beam radiation therapy (EBRT) and intermittent androgen deprivation therapy (ADT) using Goserelin acetate. Consequently, his PSA dropped down to less than 0.02 ng/mL yet rose back up to a level of 1.06 ng/mL in November 2010, i.e., seven years post operation (Figure 1). During that time, the patient was maintained on continuous ADT.

**Figure 2:**
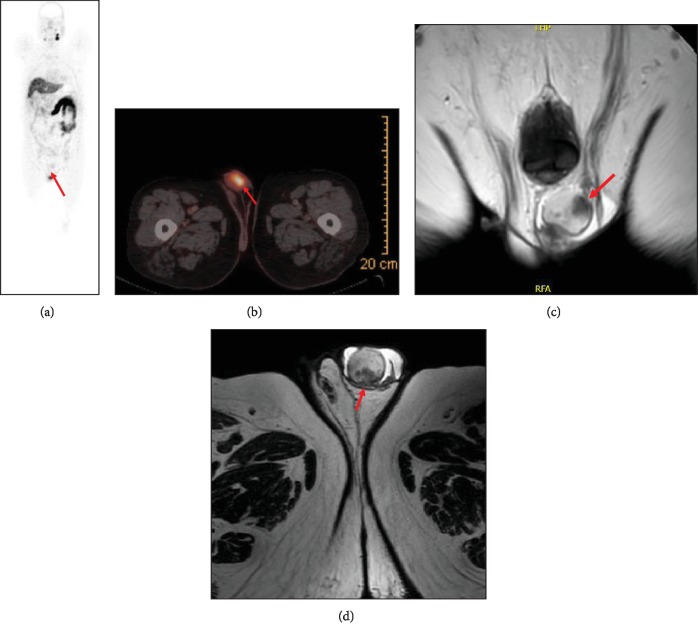
(a) ^68^Gallium prostate-specific membrane antigen-based PET maximum-intensity projection images demonstrating patient with a single left testicular metastasis (arrow); no other metastasis lesions were identified elsewhere. (b) ^68^Gallium prostate-specific membrane antigen-based PET/CT axial fusion image demonstrating again a single uptake within the left testicle, holding a SUVmax of 9.3 mSv, consistent with metastasis. (c) Coronal image of a T2-weighted (T2W) magnetic resonance image (MRI) showing several hypointense lesions involving the left testicle (arrow), suggestive of metastasis. (d) Axial image of a T2W MRI showing again several hypointense metastatic depositions within the left testicle.

Throughout the years, his PSA levels were steadily increasing; an abrupt rise in PSA to 3.9 ng/mL was noted in January 2019, sixteen years post operation (Figure 1). Physical exam at the time revealed a normal genitourinary examination along with a flat prostatic fossa upon digital rectal examination. Urine culture at the time was also negative. Upon that, a PET/CT ^68^Gallium-PSMA (prostate-specific membrane antigen) scan was performed for adequate restaging of his primary disease. A single focal radiotracer uptake was noted in the left testicle with a maximal SUV of 9.3 mSv (Figures 2(a) and 2(b)). There was no other uptake neither in the surgical bed, in the pelvic lymph nodes, nor in the bones.

A dedicated pelvic magnetic resonance imaging (MRI) was done to further characterize the testicular findings. Several poorly defined hypointense left testicular lesions were found, the largest one measuring 1.8 cm, at the level of the rete testis (Figures 2(c) and 2(d)).

**Figure 3:**
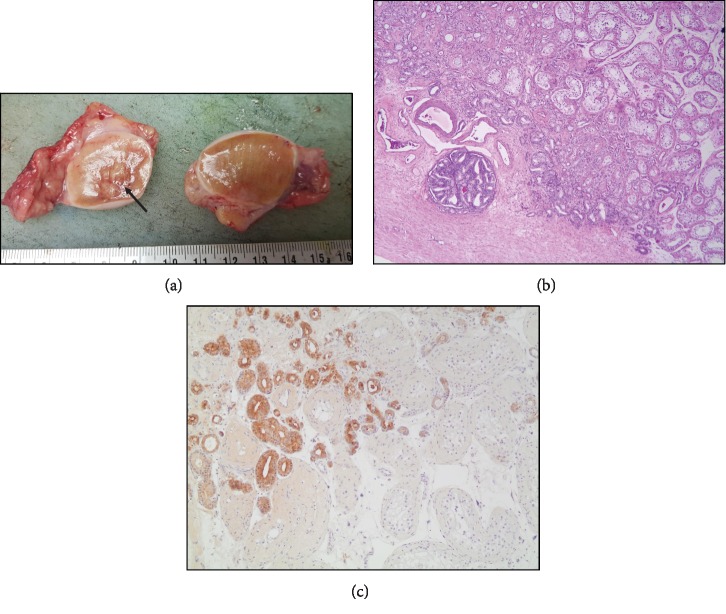
(a) Gross image of bilateral simple orchiectomy specimens revealing sectioned left testicle with multiple intraparenchymal hard nodules (arrow), ranging from 0.2 to 2.5 cm in the greatest dimension, having a tan-white hard cut surface. (b) (Hematoxylin and Eosin stain, 4x magnification) Metastatic prostate adenocarcinoma infiltrating the testicular interstitium and sparing seminiferous tubules (top right). Lymphovascular invasion in tunica albuginea (bottom left). (c) (10x magnification) Prostate-specific antigen (PSA)-positive immune-histochemical stain, highlighting the infiltration of the left testicle with metastatic prostate adenocarcinoma. The tumor is mostly made of small monolayered back-to-back acini (Gleason pattern 3). Fewer foci with cribriform glands (Gleason pattern 4) are noted. This corresponds to Gleason score 7 = 3 + 4 (grade group 2 of 5).

Following a multidisciplinary tumor board meeting, consensus was to proceed with bilateral simple orchiectomy for the purpose of surgical castration as well as pathologic examination of this left testicular lesion (Figure 3(a)). Pathology studies revealed a metastatic adenocarcinoma of prostatic origin (Figures 3(b) and 3(c)). Postorchiectomy, his PSA level dropped back to 0.02 ng/mL.

## 3. Discussion

Highlighted herein is a unique case of prostate adenocarcinoma metastasizing to the testicles 16 years post radical prostatectomy. This is a seldom seen scenario in the posttreatment phase of localized prostate cancer. An extensive literature review via PubMed, Medline, and Embase database till April 2019 revealed a total of 11 published cases [3–12]. These findings, along with our case, are summarized in Table 1. Among those cases, only two patients had a low-grade (Gleason 6) prostate cancer. Most patients had significant biochemical improvement after surgical castration. To note, all but one case presented with a preferential laterality to the left testicle. This favoritism can be attributed to mere chance, location of the primary tumor within the prostate, or the pathophysiology of prostate metastatic deposition, which is yet to be unveiled.

The most common primary tumor with testicular metastases is prostate cancer (in 15% of cases) followed by lung, kidney, and colon cancers [4]. Nevertheless, testicular metastases are rarely seen, with a reported incidence of 0.02 to 2.5%, excluding leukemia and lymphoma cases [1]. Bubendorf studied the metastatic route of prostate cancer in a cohort study involving 1589 patients diagnosed with prostate cancer upon postmortem autopsy. Over one-third of patients (35%) had metastasis via a hematogenous route. Sites of metastasis included bone in 90% of cases, followed by the lung in 45.7% of cases, while only 0.5% had isolated testicular metastasis [13].

Solid tumors metastatic to the testicles are usually very uncommon. In fact, three autopsy series, published few decades ago, have reported such incidence between 0.06% and 0.47% [14–16]. Another more recent autopsy study on 738 adult male patients with solid malignant neoplasms and mean age of 60 years showed that 5 (0.68%) patients had metastatic deposits within the testicles; the left testicle being more commonly involved, and in one (20%) case, bilateral involvement was seen [17]. The primary sites were from the lung/bronchus, melanoma of the sole, and endocrine (islet cells) carcinoma of the pancreas. The metastatic pattern is either a destructive pattern, destroying and replacing seminiferous tubules by malignant cells, or a focal interstitial pattern, characterized by distribution of tumor cells within the interstitium of the testicle without involvement of the seminiferous tubules [17].

In addition, testicular metastasis is asymptomatic and is usually diagnosed incidentally postorchiectomy for advanced prostate cancer or upon autopsy [4]. The proposed mechanisms for testicular metastasis are variable and include (1) retrograde venous extension or embolism, (2) arterial embolism, (3) lymphatic extension, and/or (4) endocanalicular spread [4]. In our case, we suggest a possible combination of all the aforementioned routes as the patient had a surgical procedure involving the prostate and the pelvic lymph nodes. We also contemplate the possibility of an additional route that is retrograde flow of tumor cells from the severed vas deferens to the testicle, given that most reports presented with seminal vesicle involvement (pT3b disease).

The distinctive aspect of our patient is the lengthy lag time between radical prostatectomy followed by adjuvant radiation and testicular metastasis, which was almost sixteen years. Lag times of previously reported cases are summarized in Table 1. Prior to our case, the longest lag time between primary treatment and testicular metastasis was 15 years where the primary prostatic disease had a Gleason score of 6 (3 + 3) with a R1 resection [7].

Such lengthy delay in presentation of metastasis within the testicles, and the rarity of such manifestation, could be attributed to the fact that the scrotum exhibits a lower temperature than the body which may be an unfavorable environment for the proliferation of tumor cells [18]. The presence of the blood-testis barrier, formed by the Sertoli cells, plays also a crucial and an indirect role in the prevention of testicular metastasis [19]. Such barrier is physiologically aimed at protecting the developing spermatozoa.

Grossly, testicular lesions secondary to metastatic prostatic adenocarcinoma show distinct nodules or large masses, well circumscribed, firm, or fleshy, and are colored yellow-white or tan. There is prominent involvement of seminiferous tubules [19], and the Gleason grade for such lesions is usually of pattern 4 or 5. The metastatic tumor mostly shows a cribriform pattern, but sometimes, a solid growth pattern or a ductal type consisting of tall columnar cells with prominent nucleoli is seen. Lymphovascular invasion is usually identified at the tumor periphery. Metastatic deposits are positive for prostate-specific antigen (PSA) staining; when negative, NKX3.1 or ERG may be helpful in such rare scenarios [19].

Prognosis of testicular metastases from a primary prostate tumor is unclear due to the rarity of the event. Some studies report a median survival of 12 months, while others report a median survival of more than 2 years [9]. Moreover, treatment strategies are yet to be defined. All reported cases opted for surgical removal of the involved testicle with a relatively good survival upon follow-up.

This case report highlights the importance of follow-up imaging after primary treatment of prostate cancer still after 16 years of treatment. Functional imaging with PET/CT ^68^Ga-PSMA is imperative as conventional imaging encompassing CT scans and bone scans would have failed to localize unusual sites of metastasis, such as the testicles.

## Figures and Tables

**Table 1:** Review of cases published of isolated testicular metastasis following primary treatment for prostate cancer.

Author (year of publication)	Gleason score	pTNM/R	PSA at diagnosis of testicular metastasis (ng/mL)	Time from treatment (years)	Laterality	PSA after orchiectomy (ng/mL)	Time of follow-up (years)
Menchini-Fabris et al. (2007) [3]	9	NA (radiation)	Undetectable	0.5	Left	Undetectable	1
Janssen et al. (2010) [4]	6 (3 + 3)	pT3N0M0R0	3.08	2.5	Left	0.07	2
Kwon et al. (2011) [5]	9 (4 + 5)	pT3NxMxR0	0.347	1.5	Right	0.03	NA
Gibas et al. (2014) [6]	7 (4 + 3)	pT2bN1R0	3.1	7	Left	0.04	1
Shinn et al. (2015) [7]	6 (3 + 3)	pT2bN0M0R1	2.98	15	Left	0.17	NA
Maibom (2017) [8]	7 (3 + 4)	cT3bN0M0Rx	4.3	2.5 (radiation)	Left	<0.1	2
Maibom (2017) [8]	9 (4 + 5)	NA	1.2	2	Left	0.1	2
Bonetta (2017) [9]	9 (4 + 5)	pT3bN0M0R1	0.61	2.5	Left	0.01	5
Cho et al. (2018) [10]	9 (4 + 5)	cT3aN1M0Rx (radiation)	2.8	4	Left	0.1	4
Presented case (2019)	7 (4 + 3)	pT3bN0M0R0	1.09	16	Left	0.02	NA

## Abbreviations

PCa: Prostate cancer
PSA: Prostate-specific antigen
TRUS: Transrectal ultrasound
EBRT: External beam radiation therapy
ADT: Androgen deprivation therapy
PSMA: Prostate-specific membrane antigen
MRI: Magnetic resonance imaging.
